# Primary Multifocal Neuroendocrine Tumor of the Breast: A Diagnostic Dilemma

**DOI:** 10.7759/cureus.100863

**Published:** 2026-01-05

**Authors:** Suyog Ratnaparkhi, Prashant Ramteke, Prashant Sawarkar, Milind Bhatkule, Pravinkumar Ghongade

**Affiliations:** 1 Pathology, All India Institute of Medical Sciences, Nagpur, Nagpur, IND; 2 General Surgery, All India Institute of Medical Sciences, Nagpur, Nagpur, IND

**Keywords:** immunohistochemistry, metastatic neuroendocrine tumor, multifocal, neuroendocrine differentiation, neuroendocrine neoplasm, primary neuroendocrine neoplasm of the breast

## Abstract

Primary neuroendocrine neoplasms (NENs) of the breast are extremely rare and diagnostically challenging tumors. We are reporting a female patient in her 40s with progressively enlarging, painless lumps measuring 30 mm and 15 mm in the upper and lower inner quadrants of the right breast for three months. Mammography reported it as the Breast Imaging Reporting and Data System (BIRADS) IVC lesion. A fine needle aspiration cytology (FNAC) done on the right breast lump and right axillary lymph node was reported as carcinoma with metastasis. Subsequent biopsy and immunohistochemistry (IHC) were reported as carcinoma with neuroendocrine differentiation. A right modified radical mastectomy with axillary lymph nodal dissection confirmed a multifocal primary neuroendocrine tumor (NET) of the breast with nodal metastasis. Accurate diagnosis of a primary NET of the breast requires clinico-radiological exclusion of a metastatic tumor. Other breast carcinomas, such as solid papillary carcinoma and hypercellular mucinous carcinoma, which are known to express NE differentiation, also need exclusion.

## Introduction

Primary neuroendocrine neoplasms (NENs) of the breast are an uncommon subtype of breast cancer originating from neuroendocrine (NE) cells within the breast tissue. Cubilla et al. [1] first described this tumor in 1977, and, over time, additional cases emerged. The World Health Organization (WHO) officially recognized them as a distinct category in 2003. Despite their rarity, accounting for less than 1% of all breast cancers, these tumors present diverse clinical and pathological characteristics that complicate diagnosis and treatment [2]. NE differentiation is typically confirmed through immunohistochemical testing for NE markers such as chromogranin A and synaptophysin [3]. However, these markers are not routinely employed for breast tumors with NE differentiation, making the actual prevalence of primary breast NENs difficult to assess. It is critical to exclude the NENs metastasis to breast and other primary breast tumors that have NE differentiation, such as the hypercellular variant of mucinous carcinoma, solid papillary carcinoma, and invasive breast carcinoma, no special type (NST) with NE differentiation. This case report will discuss the unusual presentation and diagnostic challenges of NE tumor (NET) of the breast and provide a review of the related literature.

## Case presentation

**Figure 1:**
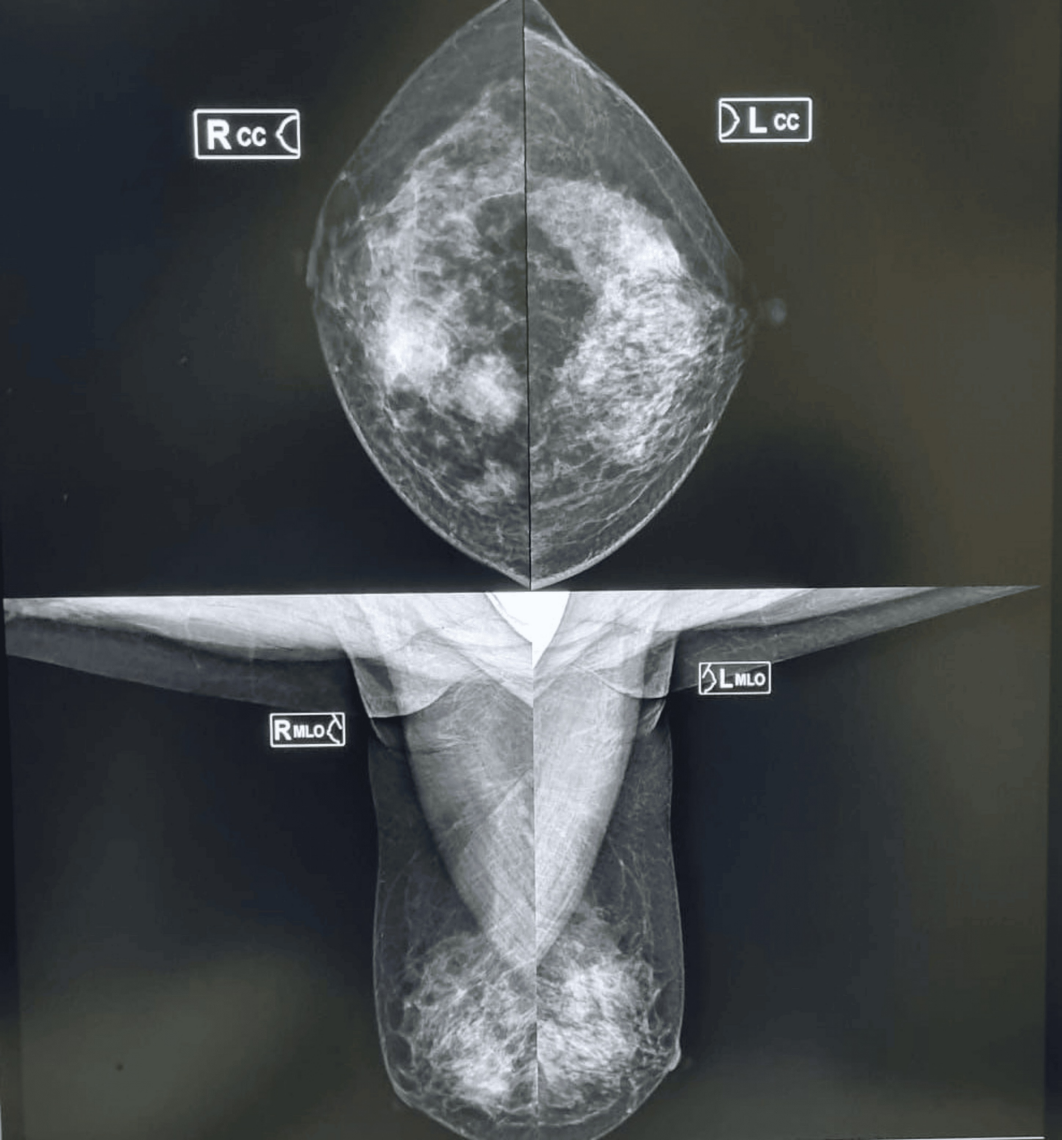
2D digital mammography Two irregular, heterogeneous, hypoechoic, solid masses measuring 30 mm and 15 mm in maximum dimension in the right breast.

A 40-year-old woman presented with a progressive mass in her right breast, which had no prior medical history or family history of cancer. Mammography revealed a Breast Imaging Reporting and Data System (BIRADS) IVC lesion with two irregular, heterogeneous, hypoechoic solid masses measuring 30 mm and 15 mm, respectively (Figure 1).

**Figure 2:**
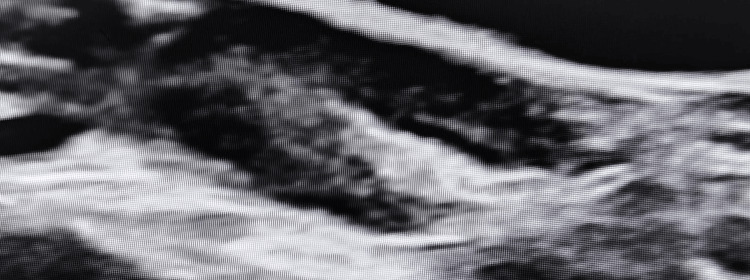
Ultrasonography of the axillary lymph node

Ultrasonography revealed a 20 mm axillary lymph node showing cortical thickening (Figure 2).

**Figure 3:**
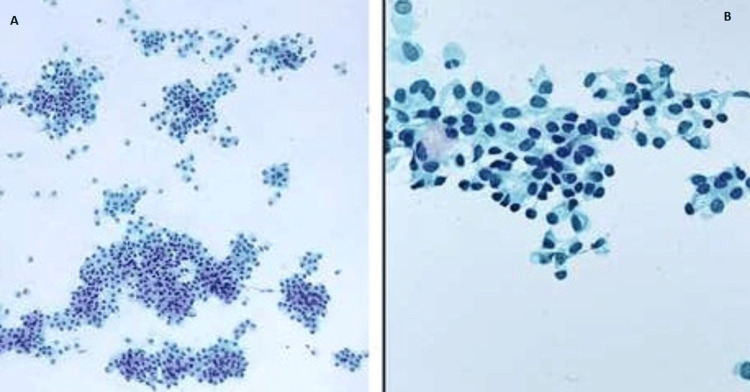
Fine needle aspiration cytology (FNAC) PAP smears (A & B) from the right breast mass shows tumor cells arranged in loose clusters, with focal acinar pattern and dispersed cells with plasmacytoid morphology, x100 and x400 respectively.

Ultrasound-guided fine needle aspiration cytology (FNAC) from the right breast lump and right axillary lymph node showed a tumor arranged in discohesive groups, singly lying, and in a focal acinar pattern. The cells were plasmacytoid and showed mild nuclear atypia (Figures 3A-3B).

The diagnosis of carcinoma was considered, with metastasis to the right axillary lymph node.

**Figure 4:**
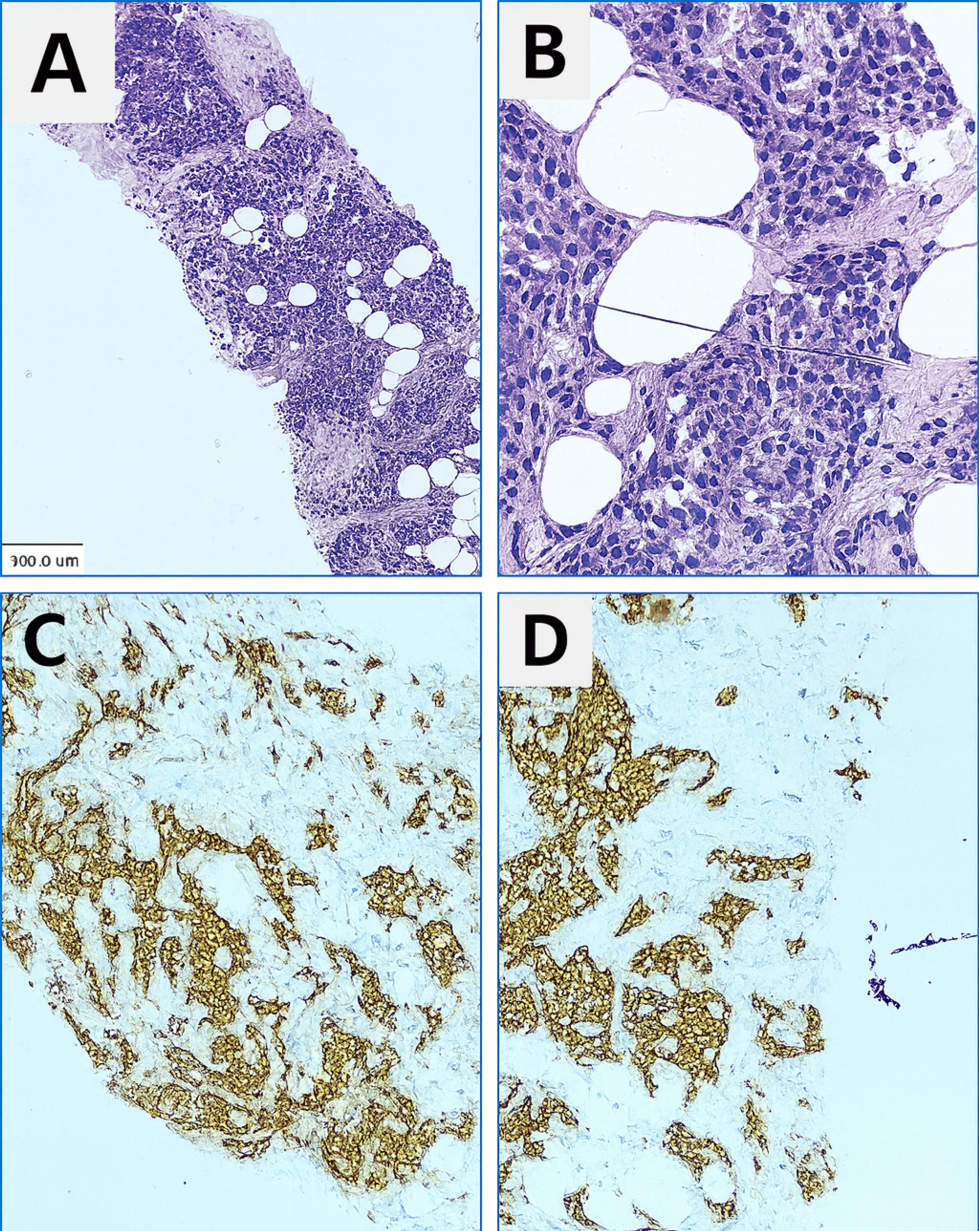
USG-guided core biopsy A & B (H&E) show the tumor in sheets and nests with eccentrically placed round to oval nucleus. C & D show tumor cells diffusely immunopositive for synaptophysin and chromogranin, respectively.

A follow-up ultrasound-guided core biopsy (Figures 4A-4D) shows a tumor in sheets and nests, which comprises monomorphic, plasmacytoid tumor cells having an eccentrically located round to oval nucleus with stippled chromatin. The tumor shows diffuse immunoexpression of synaptophysin (Syn) and chromogranin (CG) with a Ki-67 proliferation index of 2%. Estrogen receptor (ER) and progesterone receptor (PR) were strongly positive (Allred score: 8/8), while Her-2 neu was negative (0). Thus, a diagnosis of invasive carcinoma with neuroendocrine differentiation was made.

**Figure 5:**
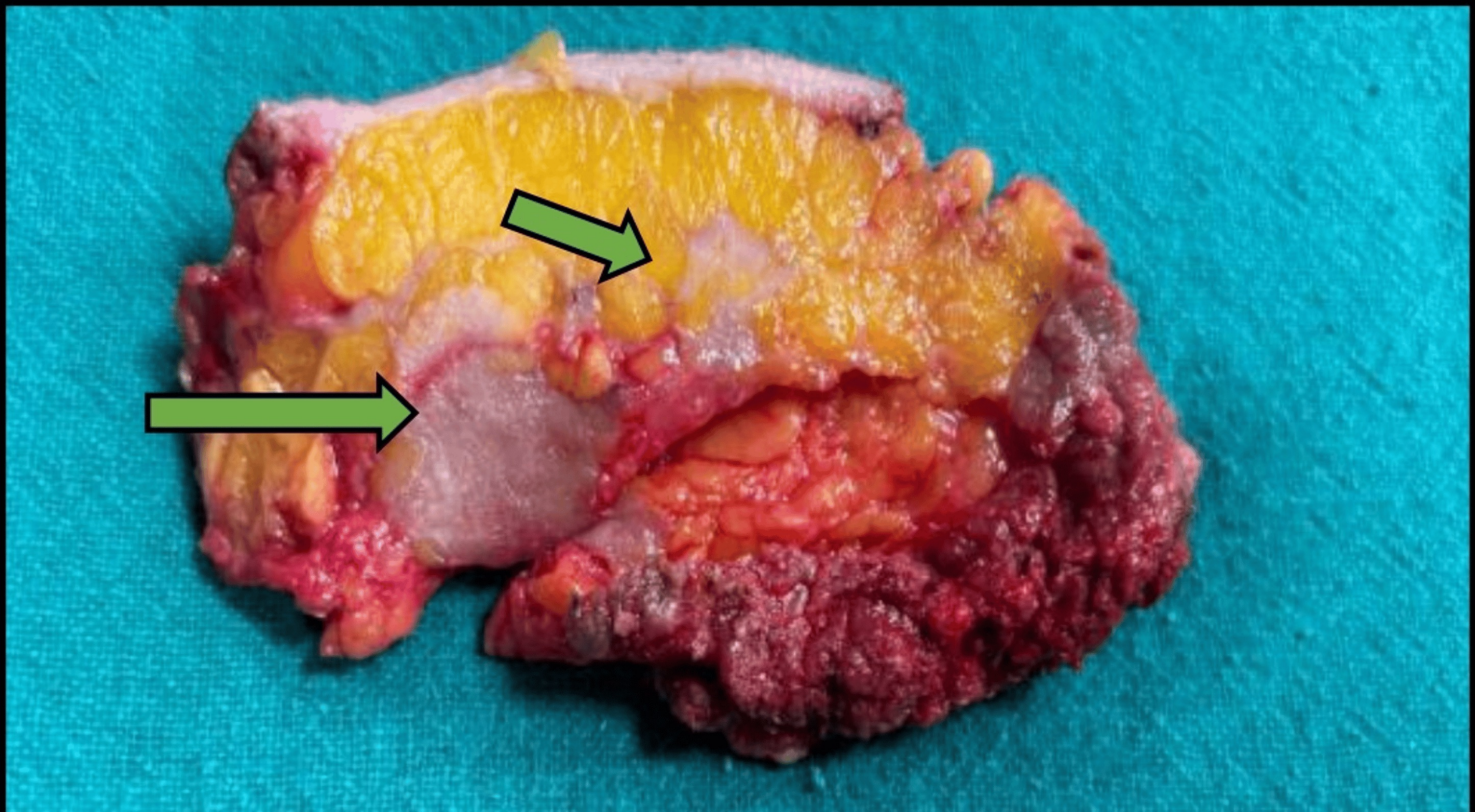
Right modified radical mastectomy gross specimen Two grey white solid tumor (green arrows), which have infiltrative edges.

The patient underwent a right modified radical mastectomy and right axillary lymph node dissection. Gross examination revealed two grey-whitish tumors measuring 30 mm and 15 mm, which were separated by > 5 mm from each other (Figure 5).

**Figure 6:**
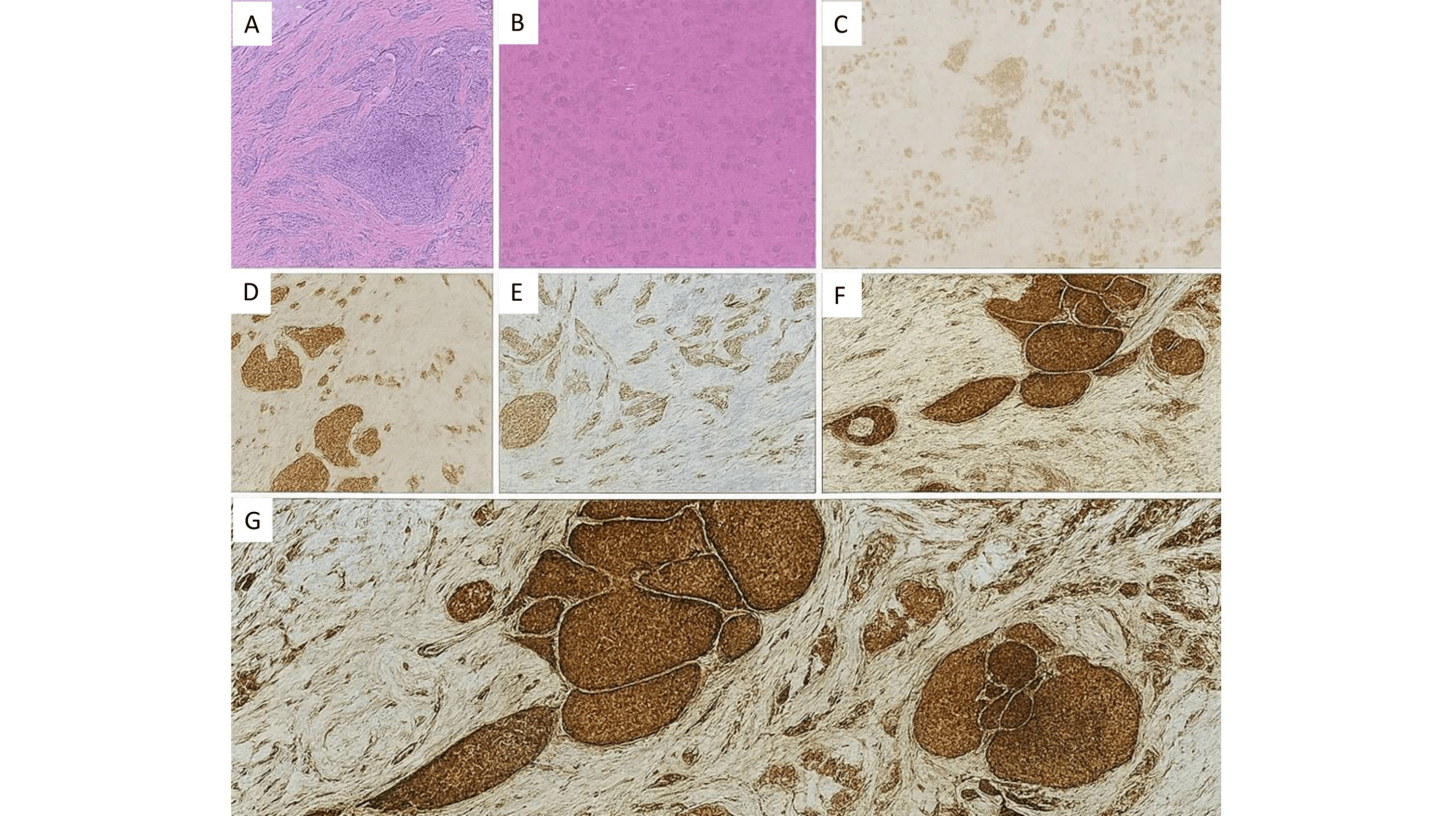
Histopathology and immunohistochemistry A: Section shows a monomorphic tumor arranged in the irregular islands separated by desmoplastic stroma and foci of ductal carcinoma in situ (DCIS), which has low nuclear grade and solid growth pattern, x40. B: Tumor cells are having round to oval nuclei with fine granular to stippled chromatin and moderate eosinophilic cytoplasm, x400. C& D: Tumor cells and the DCIS foci are diffusely immunopositive for synaptophysin and chromogranin, respectively (x40). E: Tumor cells and the DCIS foci are diffusely immunopositive for GATA3, x40. F: Tumor cells show strong cytoplasmic staining in the DCIS component, while the invasive component shows moderate cytoplasmic staining for GCDFP15, x40. G: Tumor cells are immunopsoitive for mammaglobin.

Microscopy from both the masses showed a tumor arranged in variable-sized, irregular, solid islands. The invasive tumor cells uniformly showed neuroendocrine differentiation throughout both tumors (>90%) with a prominent desmoplastic response. Areas of ductal carcinoma in situ with a solid growth pattern were noted, which had similar neuroendocrine morphology. Tumor cells were diffusely expressing Syn, CG, and a Ki-67 labeling index, which was ~2%. The tumor cells were immunopositive for GATA3, GCDFP15, and mammaglobin, while negative for TTF1 and CDX2 (Figures 6A-6G).

Three out of 17 axillary lymph nodes showed metastasis. A Ga-68 DOTANOC PET/CT scan was performed, which ruled out the presence of any other primary tumor site. Based on the overall histomorphology, immunohistochemistry, and clinico-radiological features, a diagnosis of a multifocal neuroendocrine tumor (NET) of the breast, grade 1 (pT2(m)N1a, stage IIIA) was made.

Subsequently, she received eight cycles of adjuvant chemotherapy. In the first four cycles, injection Adriamycin 90 mg and injection cyclophosphamide 900 mg were given; in the remaining four cycles, injection paclitaxel 230 mg and carboplatin 400 mg were given. Postoperatively, she has been on oral tamoxifen 20 mg to date. A follow-up PET-CT scan was done one year after the operation, revealed no evidence of malignancy, and is currently disease-free and under the oncology follow-up for two years.

## Discussion

NENs comprise less than 1% of all breast carcinomas (WHO). The current diagnostic criteria, according to the WHO blue book for breast tumors 2019, recommend classifying breast tumors with >90% neuroendocrine morphology as NENs; 10-90% as mixed invasive breast carcinoma (IBC), NST, and NEN; and <10% as invasive carcinoma NST with optional neuroendocrine pattern notation [4]. Due to inconsistent use of immunohistochemical staining and frequent changes in the WHO classification, their precise incidence remains unclear. This has also led to uncertainty regarding the clinical significance, treatment strategies, and prognosis of breast NENs.

Multicentric presentation of breast NET is infrequent and presents a diagnostic challenge, as it can be difficult to distinguish multicentric NET from metastases within the breast [5,6]. Multicentric disease adds another layer of complexity, as in one review, up to 44% (eight of 18) of metastatic NETs to the breast were misdiagnosed as primary breast tumors [7]. The distinction between primary breast NET and metastatic NET is critical to avoid misdiagnosis and unnecessary surgical and medical therapy in the latter. A thorough diagnostic work-up, including whole-body imaging, is essential to rule out metastasis from a NET arising elsewhere in the body.

Approximately 68% (15 out of 22) of primary NETs of the breast are associated with ductal carcinoma in situ (DCIS), providing strong evidence of a primary breast origin. The DCIS component was noted in the index case [8]. The most specific markers for identifying a primary breast tumor are GATA3, mammaglobin, and GCDFP15, which are usually negative in metastatic tumors. TTF1 is positive in about 70% of metastases from the lung, while CDX2 is positive in NET metastases from the lower gastrointestinal tract. Tumor cells in the index case are positive for GATA 3, GCDFP 15, and mammaglobin and are negative for TTF1 and CDX2. The other differentials for the NEN breast are IBC with NE differentiation, solid papillary carcinoma (SPC), and a hypercellular variant of mucinous carcinoma. If the NE component is 10-90%, the tumor should be classified as mixed IBC, NST, and NEN. SPC is characterized by its central/subareolar location, elderly presentation, and microscopically nodular growth with traversing fine vascular channels. Mucinous carcinoma requires > 90% tumor proportion with mucinous differentiation [9].

NE differentiation in breast carcinomas is often neglected in routine clinical practice. Tang et al. indicated that NE differentiation was not recognized in as many as 69% (51 of 74) of the breast carcinomas [10]. This high rate of oversight can be explained by the variable NE characteristics seen in primary NETs of the breast, coupled with the lack of routine testing for NE markers. Pathologists should consider ordering NE markers for further evaluation when encountering morphological features, such as monomorphic appearance, nested or trabecular pattern, classic chromatin appearance, and plasmacytoid morphology. However, it should be noted that the WHO blue book for breast tumors 2019 does not advise the routine application of NE markers to breast tumors in general due to a lack of documented clinical significance [4].

Surgical management, including mastectomy with axillary dissection and conservative surgeries, remains the primary treatment option for these tumors. Due to the rarity of breast NENs, there is no standardized chemotherapy protocol recommendation either by the National Comprehensive Cancer Network (NCCN) or by the European Society for Medical Oncology (ESMO); some clinicians treat Br-NENs like conventional breast carcinomas, while others follow the treatment protocol for NETs of the lung [11]. In the literature, we found two cases of multifocal primary breast NENs, which were diagnosed according to the 2003 WHO and 2012 WHO classifications, respectively. One case was reported as multicentric low-grade neuroendocrine carcinoma, which has an unusual tubular and cribriform pattern without axillary node metastasis. This patient underwent a quadrantectomy with wide excision, and the one-year follow-up was uneventful [6]. The other case was multicentric neuroendocrine carcinoma of the breast, which was clinically aggressive and presented with multiple metastatic lytic bone lesions. The disease responded to systemic therapy with an oral aromatase inhibitor and monthly bisphosphonate infusions [5]. The index case was treated as per the recommendation of NCCN guidelines for invasive breast carcinoma (T2) with the positive hormone receptor status, HER2-negative status, and axillary lymph nodal metastasis.

The prognosis of primary NEN of the breast primarily depends on the tumor’s histological grade and clinical stage [12,13]. These tumors should be graded according to the Nottingham Grading System as per the WHO breast tumor recommendation [4]. The index case was graded as grade 1. These tumors tend to progress slowly, similar to NET in other organs [12]. Yang et al. found that the five-year disease-specific survival (DSS) rates for NETs, neuroendocrine carcinomas (NECs), and invasive ductal carcinoma not otherwise specified (IDC-NST) were 63.39%, 46.00%, and 89.17%, respectively. The five-year overall survival (OS) rates for NETs, NECs, and IDC-NST were 55.66%, 38.87%, and 83.17%, respectively. NETs and NECs demonstrated significantly lower DSS and OS than corresponding stage or grade IDC-NST tumors (all p < 0.050) [14]. In the index case, there is no evidence of local recurrence or metastasis on routine follow-up for the last two years.

## Conclusions

Primary multifocal NET of the breast is an exceptionally rare entity and should be diagnosed only when the tumor shows neuroendocrine marker expression in more than 90% of the tumor cells and after excluding metastatic disease. The presence of an associated DCIS component serves as a crucial indicator of primary breast origin. Accurate diagnosis relies on extensive tumor sampling, detailed histopathological evaluation, immunohistochemistry, and comprehensive radiologic work-up. Further research involving larger patient cohorts is needed to better define the biologic behavior and prognostic implications and to establish standardized diagnostic and management protocols for these rare tumors.
